# Exploration of Shared Genetic Architecture Between Subcortical Brain Volumes and Anorexia Nervosa

**DOI:** 10.1007/s12035-018-1439-4

**Published:** 2018-12-05

**Authors:** E. Walton, D. Hibar, Z. Yilmaz, N. Jahanshad, J. Cheung, V.-L. Batury, J. Seitz, C. M. Bulik, PGC-ED, ENIGMA Genetics Working Group, P. M. Thompson, Stefan Ehrlich

**Affiliations:** 1 MRC Integrative Epidemiology Unit, Population Health Sciences, Bristol Medical School, University of Bristol, Bristol, UK; 2 Division of Psychological and Social Medicine and Developmental Neurosciences, Faculty of Medicine, Technische Universität Dresden, Fetscherstr. 74, 01307 Dresden, Germany; 3 Imaging Genetics Center, Mark and Mary Stevens Neuroimaging & Informatics Institute, Keck School of Medicine of the University of Southern California, Marina del Rey, Los Angeles, CA USA; 4 Janssen Research & Development, San Diego, CA USA; 5 Department of Psychiatry, University of North Carolina at Chapel Hill, Chapel Hill, NC USA; 6 Department of Genetics, University of North Caroline at Chapel Hill, Chapel Hill, NC USA; 7 Department of Child and Adolescent Psychiatry, Psychotherapy and Psychosomatics, University Hospital RWTH University Aachen, Aachen, Germany; 8 Department of Medical Epidemiology and Biostatistics, Karolinska Institutet, Stockholm, Sweden; 9 Department of Nutrition, University of North Carolina at Chapel Hill, Chapel Hill, NC USA

**Keywords:** Anorexia nervosa, Brain structure, Genetic correlation

## Abstract

In MRI scans of patients with anorexia nervosa (AN), reductions in brain volume are often apparent. However, it is unknown whether such brain abnormalities are influenced by genetic determinants that partially overlap with those underlying AN. Here, we used a battery of methods (LD score regression, genetic risk scores, sign test, SNP effect concordance analysis, and Mendelian randomization) to investigate the genetic covariation between subcortical brain volumes and risk for AN based on summary measures retrieved from genome-wide association studies of regional brain volumes (ENIGMA consortium, *n* = 13,170) and genetic risk for AN (PGC-ED consortium, *n* = 14,477). Genetic correlations ranged from − 0.10 to 0.23 (all *p* > 0.05). There were some signs of an inverse concordance between greater thalamus volume and risk for AN (permuted *p* = 0.009, 95% CI: [0.005, 0.017]). A genetic variant in the vicinity of ZW10, a gene involved in cell division, and neurotransmitter and immune system relevant genes, in particular *DRD2*, was significantly associated with AN only after conditioning on its association with caudate volume (p_FDR_ = 0.025). Another genetic variant linked to *LRRC4C*, important in axonal and synaptic development, reached significance after conditioning on hippocampal volume (p_FDR_ = 0.021). In this comprehensive set of analyses and based on the largest available sample sizes to date, there was weak evidence for associations between risk for AN and risk for abnormal subcortical brain volumes at a global level (that is, common variant genetic architecture), but suggestive evidence for effects of single genetic markers. Highly powered multimodal brain- and disorder-related genome-wide studies are needed to further dissect the shared genetic influences on brain structure and risk for AN.

**Electronic supplementary material:**

The online version of this article (10.1007/s12035-018-1439-4) contains supplementary material, which is available to authorized users.

## Introduction

Anorexia nervosa (AN) is an often life-threatening, adolescent-onset eating disorder characterized by severe emaciation, and typically by severe food restriction. AN mortality rates are the highest in psychiatry [1].

Despite high twin-based heritability estimates of around 50**–**80% for AN [2], we do not fully understand the pathophysiology of the disorder. So far only one common variant on chromosome 12 and no rare variants above genome-wide thresholds have been detected in four studies based on up to 3500 AN cases and 15,000 controls [3–6].

In acutely ill patients with AN, reduction of brain tissue is often readily visible in individual patients’ brain scans. A number of structural MRI studies (albeit with small sample sizes) have documented decreases in both gray and white matter volumes (for review, see ref. [7, 8]). More recent, larger studies and a meta-analysis have confirmed widespread gray matter volume reductions in AN, with regional effects especially in reward-related and somatosensory areas [9–11]. In weight-recovered patients, subcortical and cortical gray matter deficits seemed to normalize, but several studies have reported small group differences even after recovery [9, 12–14].

In light of these structural abnormalities, one question is whether certain structural brain differences share a similar genetic architecture with AN and may possibly even reflect a predisposition for AN. Magnetic resonance imaging (MRI) measures of brain structure are moderately heritable [15] and several volumetric measures have high reproducibility and low measurement error [16]. Ideally, an answer to the above question might require longitudinal, prospective population-based studies with brain scans in healthy individuals who will go on to develop AN later in life. Considering the high cost, low power, and logistical challenges of such a study, a more feasible alternative approach would be to examine the genetic overlap between AN and brain structure. Unfortunately, the number of existing genetic and neuroimaging studies in AN is relatively small compared to other major neuropsychiatric disorders. To date, only two imaging genetics studies have been published [17, 18], suggesting that *COMT* and *5-HTTLPR* genotype may modulate functional connectivity in AN patients.

We are now able to leverage results from large-scale, genome-wide association studies (GWAS) based on tens of thousands of individuals. Data from GWAS on brain structure and on genetic risk for AN allows us to investigate the genetic covariation between brain structure and disease risk. Investigating this genetic correlation should inform us about shared genetic influences between brain structure and AN. A large degree of genetic overlap could indicate potential pleiotropic effects, where the same genetic variants influence both traits. Results could then help to derive or adapt hypotheses about how brain structure is involved in AN etiology. Several groups have investigated genetic overlap between structural brain measures and risk for schizophrenia as well as other psychiatric disorders [19–22].

In the current study, we followed a roadmap for the analysis of genetic covariation between brain volumes and AN using a battery of complementary methods including those suggested by Franke et al. [20]. To date, well-powered summary estimates are available for eight structural brain measures (intracranial volume (ICV) and seven subcortical regions) from published GWAS [16]. Therefore, our analysis focused on ICV and these regional subcortical volumes. In detail, we investigated the potential for a shared genetic architecture based on common genetic variation as well as of an overlap of individual genetic risk variants between both disorder and brain measures.

## Materials and Methods

In this article, we used independent data from separate GWAS on brain structure and on genetic risk for AN to study the genetic covariation between these measures.

### Samples

#### Subcortical Brain Volume GWAS Summary Statistics from the Enhancing NeuroImaging Genetics Through Meta-Analysis Consortium (ENIGMA)

ENIGMA MRI summary measures from genetic association analyses of ICV and seven subcortical volumes [16] were available online at http://enigma.usc.edu/research/download-enigma-gwas-results/. These analyses were based on brain MRI scans and genome-wide genotype data for 13,170 subjects from 28 cohorts Online Resource, section 1.1 and Table S1). All participants in all cohorts in this study gave written informed consent and sites involved obtained approval from local research ethics committees or Institutional Review Boards. In the original analysis, GWAS statistics from each of the 28 sites had been combined using a fixed-effect inverse variance-weighted meta-analysis as implemented in METAL [23].

#### Anorexia Nervosa GWAS Summary Statistics from the Eating Disorders Working Group of the Psychiatric Genomics Consortium (PGC-ED)

Anorexia nervosa (AN) cases met DSM-IV criteria for either lifetime AN (restricting or binge-purge subtype) or lifetime eating disorders ‘not otherwise specified’ AN-subtype (i.e., exhibiting the core features of AN) [24]. Summary measures were available on genetic association analyses on AN diagnosis (https://www.med.unc.edu/pgc/results-and-downloads) [4]. These analyses were based on AN phenotype and genome-wide genotype data for 3495 AN cases and 10,982 control subjects from 12 cohorts [3, 6] (Table S2). To our knowledge, PGC-ED AN cohorts did not overlap with cohorts in the current ENIGMA GWAS. Participants clustered with subjects of known European ancestry. Genomic data were imputed to a reference panel (1000 Genomes, phase 3), using SHAPEIT [25] for phasing and IMPUTE2 for imputation [26]. Tests of AN association within datasets were performed using an additive model in PLINK [27] with the first ten principal components as covariates. Fixed-effects meta-analysis across the 12 datasets was carried out using METAL [23] with inverse variance weighting. For more information, see Online Resource section 1.2 and reference [4].

### Statistical Analysis

#### Linkage Disequilibrium Score Regression

Linkage disequilibrium score (LDSC) regression [28] was used to assess genome-wide common variant heritability and genetic correlations between AN and subcortical volumes. In detail, LDSC assesses whether inflation in GWAS test statistics is due to polygenicity or other confounding biases such as cryptic relatedness or population stratification. For this analysis, each dataset was filtered to only include markers overlapping with HapMap Project Phase 3 SNPs (N_overlap_ = 1,161,164), as these tend to be well-imputed across studies and alleles will match those listed in the data used to estimate the LD score. No SNPs had out-of-bound *p* values or were strand-ambiguous. Because ENIGMA subcortical brain volume and PGC-ED AN measures were based on European samples, we used pre-computed LD scores for European populations, as provided on the LDSC website (https://github.com/bulik/ldsc). Standard errors were estimated using a block jackknife procedure and used to calculate *p* values. *p* values were adjusted for seven tests (eight brain regions minus the amygdala; see Franke et al., [20] and Results below) to account for multiple testing.

#### Genetic Risk Score Analysis

We used the grs.summary function developed by Johnson [29] and implemented in PRSice (version 1.25, ref. [30]), which approximates the regression of a response variable (i.e., risk for AN based on PGC-ED GWAS) onto an additive multi-SNP genetic risk score. Risk score coefficients are weighted by single SNP regression coefficients estimated from one set of GWAS results (here: ENIGMA subcortical brain volume). We investigated the effect at four *p* value thresholds (1*10^−4^, 1*10^−3^, 1*10^−2^, 5*10^−2^) and adjusted for 28 tests (eight brain regions minus the amygdala * four thresholds) to account for multiple testing.

#### Sign Test

We employed a sign test as an additional method to investigate a potential overlap of positive or inverse direction effects of SNPs between both datasets at *p* value thresholds (1*10^−4^, 1*10^−3^, 1*10^−2^, 5*10^−2^). Using the binom.test function from the stats package in R, we tested the significance of the number of SNPs with opposite direction effects between datasets at these four thresholds over the total number of SNPs. *p* values were adjusted for 28 tests (eight brain regions minus the amygdala * four thresholds) to account for multiple testing.

#### SNP Effect Concordance Analysis

SNP effect concordance analysis (SECA) tests for pleiotropy, concordance, and ‘pleiotropy-informed’ conditional false discovery rate (FDR) results between two sets of GWAS summary results [31]. SECA estimates whether (a) there is an excess of SNPs associated with the respective phenotype in both datasets (pleiotropy); (b) the directions of effect are in agreement across datasets (concordance); and (c) single SNPs in dataset 2 (here: PGC-ED AN) gain in significance after conditioning on their strength of association in dataset 1 (here: ENIGMA subcortical brain volumes; conditional results). Concordance analysis in SECA tests for positive concordance (i.e., whether a larger OR_AN_ relates to a larger BETA_subcortical_). However, since we were interested in the opposite relationship between these variables (i.e., whether a OR_AN_ greater than one relates to a negative BETA_subcortical_), we derived and used the inverse of the OR_AN_ in the concordance analysis. For all SECA analyses, overlapping SNPs between both datasets (*N* = 7,868,363) were pruned for LD using a *p* value informed method, a 1 Mb window and r^2^ > 0.1 (all default settings in SECA). This resulted in *N* = 26,558 SNPs, which were entered in the analysis. *p* values were adjusted for seven tests (eight brain regions minus the amygdala; see Franke et al., [20] and Results below) to account for multiple testing.

#### Mendelian Randomization

To investigate potentially causal relationships between subcortical brain volumes and AN, we applied a two-sample Mendelian Randomization (MR) approach, which only requires GWAS summary level data. MR is a method of investigating causal relationships by using genetic variants as instrumental variables [32]. The main assumptions and strengths of the technique have been outlined in detail elsewhere [33–35]. Briefly, to select SNPs that are strong instrumental variables (relevance assumption), we investigated only brain volumes and SNPs where the genetic variants associated with brain volume at a genome-wide level significant level (caudate (1 SNP), hippocampus (2 SNPs), putamen (4 SNPs, of which 3 were available in the AN GWAS summary data), and ICV (1 SNP); as reported in Hibar et al. [16]). We were not able to investigate the causal effect of AN on brain volumes, as the AN-linked variant (rs4622308) was not available in the subcortical GWAS summary data. Due to the limited number of strongly associated genetic variants per structure, we used the Wald ratio method and hence were unable to investigate presence of horizontal pleiotropy as a potential violation of the MR exclusion restriction assumption. To limit confounding due to population stratification (a potential violation of the independence assumption), we used GWAS summary data based on largely European populations. The TwoSampleMR package in R (also available as part of the MR-Base (www.mrbase.org) platform [36] was used for all MR analyses.

## Results

The following analyses were based on summary statistics for (a) eight brain volume measures of 13,170 participants from the ENIGMA consortium and (b) AN case-control data from 3495 AN patients and 10,982 healthy individuals. We focused on ICV and all subcortical regions (caudate, hippocampus, pallidum, nucleus accumbens, putamen, thalamus, amygdala) that were investigated in [16], and hence had GWAS summary data available.

### Linkage Disequilibrium Score Regression

Linkage disequilibrium score (LDSC) regression examines the relationship between two sets of GWAS test statistics using linkage disequilibrium. Restricting our analyses to Hapmap3 SNPs (as recommended, see methods) left 1,161,164 SNPs in the ENIGMA datasets and 1,217,311 in the AN dataset with a total of 1,094,348 overlapping SNPs. SNP-based heritability estimates for the traits were 26% (caudate, 95% CI [18; 34]), 15% (hippocampus [95% CI 7; 23] and pallidum [95% CI 7; 24]), 9% (nucleus accumbens, 95% CI [1; 17]), 31% (putamen; 95% CI [21; 41]), 14% (thalamus; 95% CI [6; 22]), 18% (ICV; 95% CI [9; 27]), and 23% (AN; 95% CI [16; 31]; Table 1). The GWAS-estimated heritability for the amygdala volume was not significantly different from zero (− 2%, 95% CI [− 9; 4]). This structure was therefore omitted from the following analyses. Using LDSC regression, we did not identify a significant genetic correlation between any of the remaining seven brain volumes and AN (Table 1).

**Table 1:** Genetic overlap analyses between anorexia nervosa (AN) and seven subcortical brain volumes

ROI	LDSC					GRS^a^			Sign test^a^				SECA	
Volume	h^2^ in %	95% CI	rg	se	p(rg)	R^2^	p(GRS)	p threshold (brain volume)	Estimate^b^	95% CI	p(binomial)	p threshold (brain volume)	p(pleiotropy)	p(concordance)
Caudate	25.65	[17.61,33.69]	0.14	0.11	0.19	9.55E-05	0.120	1E-04	0.513	[0.49,0.535]	0.268	1E-02	1.000	1.000
Hippocampus	14.86	[6.61,23.11]	− 0.06	0.15	0.66	3.42E-04	0.013^c^	1E-02	0.522	[0.499,0.544]	0.061	1E-02	1.000	0.138
Nucleus accumbens	8.93	[0.76,17.1]	0.14	0.19	0.45	1.43E-04	0.075	1E-03	0.450	[0.384,0.518]	0.159	1E-03	0.057	0.052
Pallidum	15.25	[6.84,23.66]	0.10	0.14	0.47	2.65E-04	0.025^c^	1E-02	0.522	[0.499,0.545]	0.065	1E-02	1.000	0.311
Putamen	30.80	[20.78,40.82]	0.05	0.10	0.61	1.26E-04	0.089	1E-04	0.438	[0.374,0.503]	0.061	1E-03	0.153	0.170
Thalamus	13.74	[5.78,21.7]	− 0.10	0.15	0.49	2.23E-04	0.036^c^	5E-02	0.512	[0.502,0.523]	0.019^d^	5E-02	0.160	0.009^c,d^
ICV	17.96	[8.59,27.33]	0.23	0.14	0.10	3.28E-04	0.015^c^	1E-03	0.560	[0.495,0.623]	0.072	1E-03	1.000	0.078

*ROI*, region of interest; *ICV*, intracranial volume; *LDSC*, LD score regression; *GRS*, genetic risk score; *SECA*, SNP effect concordance analysis; amygdala volume was omitted from the analyses because of low heritability; heritability estimates on the observed scale; rg = genetic correlation with AN; p(rg) = *p* value of the genetic correlation; se = standard error; CI = confidence interval; ^a^ tests were carried out on subsets of four different thresholds (1*10^−4^, 1*10^−3^, 1*10^−2^, 5*10^−2^) in the discovery dataset (i.e., ENIGMA subcortical brain volume), but only the strongest results are reported in the table. For detailed results, see Tables S3 and S4. ^b^ An estimate > 0.5 indicates a higher-than-chance proportion of SNPs with a risk effect on brain volume (BETA<0) and on AN (OR > 1); ^c^ nominally significant at *p* < 0.05; ^d^ trend significance after Bonferroni-correction for 7 tests (number of brain volumes)

### Genetic Risk Score Analysis

**Fig. 1:**
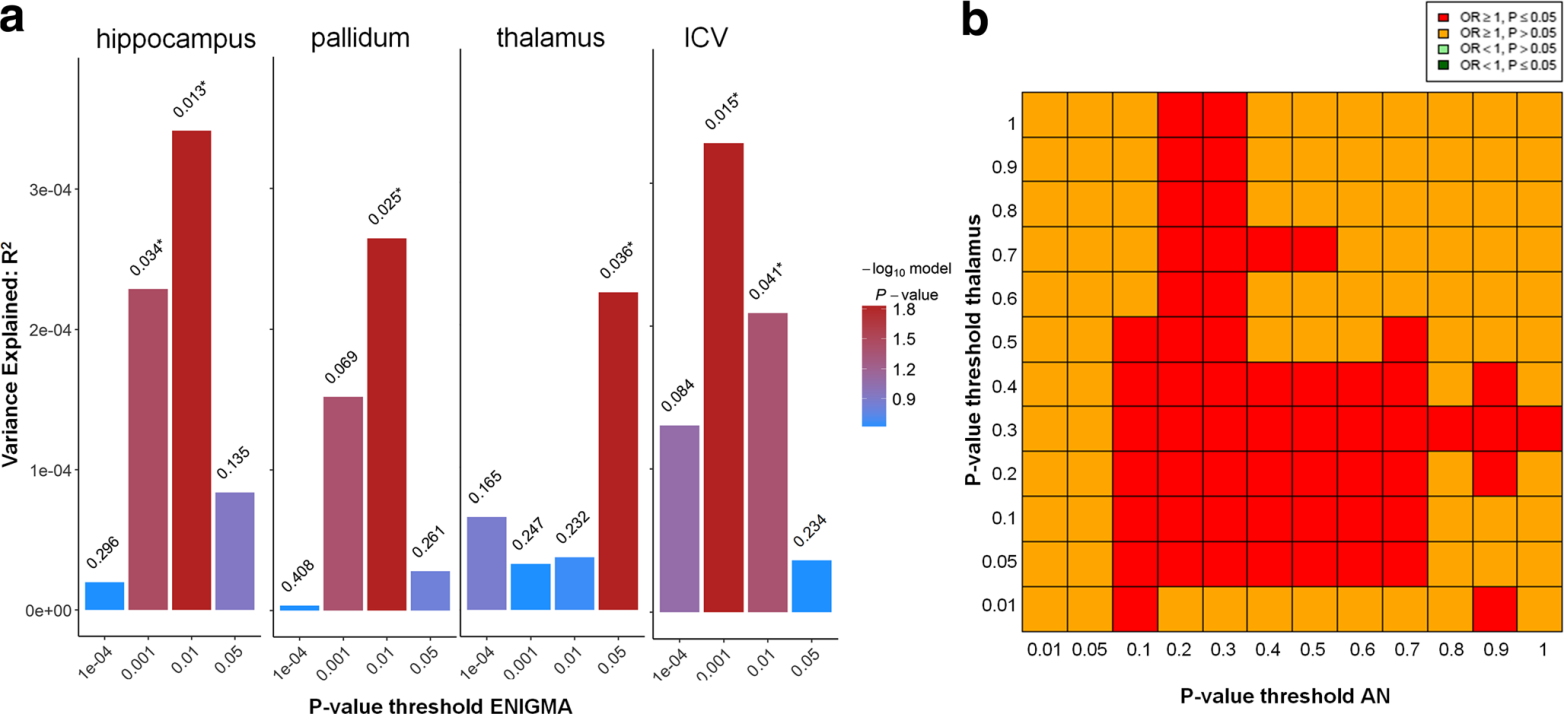
**a** Genetic risk score analysis results for four subcortical brain volumes with nominally significant results. *p* value criteria used to threshold ENIGMA input data are plotted on the x-axis, the amount of variance of AN liability explained (R^2^) on the y-axis. The color bar indicates the level of significance for a GRS effect on AN. **b** SECA analysis indicated significant concordance effects between AN (x-axis) and thalamus volume (y-axis). For computational purposes, OR_AN_ was inversely coded, so that red indicates concordance of SNP effects between increased risk for AN and lower thalamus volume

Next, we tested the effect of additive multi-SNP genetic risk scores, weighted by ENIGMA subcortical brain volume betas, onto PGC-ED AN, to see whether risk for altered subcortical brain volume is related to risk for AN. No effects were observed after correcting for 28 tests (seven brain regions * four thresholds). For four brain regions (hippocampus, pallidum, thalamus, and ICV), nominal effects were observed, but the amount of explained variance was negligible (less than 0.034%). See Table 1, Fig. 1a and Table S3 for further details.

### Sign Test

Testing for an accumulation of positive or negative direction effects of SNPs in the PGC-ED AN and ENIGMA datasets at four different ENIGMA *p* value thresholds (1*10^−4^, 1*10^−3^, 1*10^−2^, 5*10^−2^), we could not identify any significant effects after adjusting for multiple testing. There was a nominally significant effect (p_threshold < 0.05_ = 0.019) for the thalamus, where we found a higher proportion of SNP alleles with a negative effect on brain volume (BETA < 0) and an increased risk for AN (OR > 1) than would be expected by chance. This effect was not found when restricting the analyses to SNPs passing thresholds based on the AN GWAS results instead. See Table 1 and Table S4 for further details.

### SNP Effect Concordance Analysis

SNP effect concordance analysis (SECA) extracts subsets of independent SNPs (present in both datasets) to test at 12 different *p* value thresholds (resulting in 144 subsets) for an excess of SNPs associated in both datasets (*pleiotropy*), for *concordance* of effect directions, and for *conditional effects*. After LD-pruning, 26,558 SNPs were left in the analysis. There was an overall effect for an inverse concordance between the thalamus and AN (i.e., SNP effects related to smaller thalamus volume and larger OR_AN_), which remained significant only at a trend level after correcting for seven tests (uncorrected *p*_concordance_ = 0.009; Table 1 and Fig. 1b). There were no other signs of pleiotropy or concordance between any of the remaining volumes and AN (Table 1). Testing for conditional effects, we identified individual SNPs that gained in association significance with AN after conditioning on their strength of association with subcortical volume for the caudate, hippocampus, nucleus accumbens and the pallidum. In detail, rs3863294 on chromosome 11—located close to the genes *ZW10* (involved in cell division) and *TMPRSS5* (highly expressed in brain tissue) and also in the vicinity of neurotransmitter and immune system relevant genes such as *DRD2*, *HTR3B*, *HTR3A*, and *NCAM* (Fig. S1)—was significantly associated with AN only after conditioning on its association with caudate volume (p_FDR-noCond_ = 0.324 to p_FDR-caudCond_ = 0.0246). Follow-up analyses on functional effects of rs3863294 on DNA methylation or gene expression indicated an influence on DNA methylation linked to *ZW10* in blood, but not in brain tissue and an effect on gene expression of *TMPRSS5* in brain tissue and of *DRD2* in peripheral tissue (Online Resource section 2.4).

Similarly, rs7945461—linked to *LRRC4C* which is important in axonal and synaptic development—gained significance after conditioning on hippocampal volume (p_FDR-noCond_ = 0.272 to p_FDR-hipp-Cond_ = 0.0208). Two SNPs that were conditional on volumes of the nucleus accumbens and the pallidum (rs6708971; p_FDR-noCond_ = 0.127 to p_FDR-accumb-Cond_ = 0.009; and rs873507; p_FDR-noCond_ = 0.093 to p_FDR-pall-Cond_ = 0.034) were located in intergenic regions. For further details on these conditional analyses, see Online Resource section 2.5.

### Mendelian Randomization

To assess potential causal effects of brain volume on AN, genetic variants linked to each of the four brain volumes with genome-wide level markers were used as instruments. Wald ratios did not indicate causal effects of any brain volume on risk for AN (Table S5).

## Discussion

We evaluated the relationship between common genetic variants implicated in subcortical brain volumes and those associated with a clinical diagnosis of AN. The sample sizes were the largest yet applied to these questions. With a comprehensive set of analyses, we found weak evidence for strong genetic correlations at a global level (that is, common variant genetic architecture) and suggestive evidence for effects of single genetic markers.

There were signs of an inverse concordance between thalamus volume and risk for AN (i.e., SNP effects related to smaller thalamus volume and larger OR_AN_). For individual genetic variants, we identified a variant in the vicinity of neurotransmitter, development and immune system relevant genes such as *ZW10*, *TMPRSS5* and *DRD2*, *HTR3B*, *HTR3A*, and *NCAM* to be significantly associated with AN only after conditioning on its association with caudate volume. Another variant linked to *LRRC4C*, important in axonal and synaptic development, gained significance after conditioning on hippocampal volume.

Several reasons could explain why we did not detect a major genetic overlap between AN and subcortical volumes in the current study. In this study, we focused on volume alterations in subcortical regions which are potentially implicated in AN, but other neuroimaging phenotypes, such as measures of cortical thickness or surface area as well as resting-state functional or anatomical connectivity, may also be informative. Similarly, genetic risk for AN might relate to specific cell types or structures that do not easily relate to those properties detected by the structural imaging approach applied in this study. Second, it is possible that the samples in the PGC-ED GWAS are somewhat heterogeneous with respect to disease severity or subtype. More robust differences might be found when larger sample sizes and deeply phenotyped data allow us to focus on, e.g., subtype effects (restrictive versus binge-purge) or select individuals with more severe and enduring psychopathologies or those with particularly high genetic risk for AN. Third, the data analyzed in this study are largely based on adult samples. Heritability estimates vary with age and the degree of genetic overlap between subcortical volume and AN may be larger in younger populations. Fourth, we evaluated only common genetic variation. It is possible that rare variants play a role in the shared genetic architecture between brain volume and AN. Last but not least, it is indeed possible that there is no link between the genetics of subcortical volumes and AN. However, the results of the conditional analysis draw such a conclusion into question.

Our results are similar to other studies that reported a lack of a shared genetic basis between brain volumes and schizophrenia as well as major depression [20, 22]. However, another recent study reported an enriched contribution of schizophrenia GWAS loci to intracranial volume (a global measure of brain morphology; Lee et al. [21]). The latter study also included several other psychiatric traits as part of an exploratory analysis and found no associations between risk for AN and intracranial volume. It is likely that genetic variants that are linked to complex disorders such as AN may exert their effects through various biological pathways affecting different systems to varying degrees. This high level of heterogeneity might prevent us from identifying more distinct genetic signals on brain volume. Alternative methods such as those described in Smeland et al. [37] and Lee et al. [21], or parallel ICA [38], some of which are able to delineate independent genetic signals on distinct brain networks might provide a potentially promising approach to study the relationship between brain volume and AN.

Despite our negative findings, several studies have reported genetic correlations between AN and different clinical traits. A significant twin-based genetic correlation between AN and OCD has been observed using data from the population-based Swedish Twin Registry (additive genetic correlation = 0.52; ref. [39]) and has since been replicated with a SNP-based genetic correlation by the PGC consortium (r_g_ = 0.53; ref. [40]). A recent study [4] has found positive genetic correlations between AN and a range of psychiatric traits including schizophrenia and neuroticism, perhaps reflecting genetic risk for general psychopathology. Negative genetic associations were observed with several “unfavorable” metabolic phenotypes (such as fasting insulin, fasting glucose or insulin resistance), suggesting that metabolic factors might be involved in dysregulation of weight and appetite in AN.

We detected an inverse concordance between genetic determinants of thalamus volume and AN. Gray matter atrophy in the thalamus in AN has been reported in previous studies [10, 14] and diffusion-based MRI investigations have frequently implicated alterations of white matter tracts connecting the thalamus and especially fronto-parietal regions with AN [41–43]. Studies using functional MRI have also found differences in thalamus functioning in AN [44], indicating that this region might be involved in motivation-related behavior in AN. Moreover, thalamo-frontal circuit abnormalities in AN (measured using an fMRI-based resting-state functional connectivity approach) were linked to cognitive control and working memory performance in patients [45]. Reduced local thalamic network efficiency (indicated through decreased connectivity strength and increased path length) in patients with AN was further supported using an approach that modeled the entire brain as a complex network [46, 47]. These results are in line with a model for human awareness and subjectivity by Craig et al. [48], which postulates that afferent representations of the physiological state of the body ascend from the spinal cord via the brain stem and the thalamus to the insula. Therefore, reduced connectivity in a network including the thalamus might reflect a modified calibration of signals such as body size or hunger and may subsequently contribute to typical AN symptoms. Such abnormalities in signal processing might be genetically determined, arise during neurodevelopment and manifest in altered thalamus volumes and connectivity. However, considering largely trend level effects in our analyses, as well as the fact that we could not investigate causal associations through Mendelian randomization due to the absence of strong genetic loci associated with thalamus volume, we cannot draw any conclusions about the causality or direction of effect (i.e., whether reduced thalamus volume is a risk factor or consequence of AN or whether this association is due to confounding).

We also found that genetic variant rs3863294, located close to the genes *ZW10* (involved in cell division) and *TMPRSS5* (highly expressed in brain tissue) and also in the vicinity of neurotransmitter and immune system relevant genes such as *DRD2*, *HTR3B*, *HTR3A*, and *NCAM*, was significantly associated with AN after conditioning on its genetic association with caudate volume. A direct link between rs3863294 and these genes deserves further investigation. However, it is interesting to note that prior candidate gene studies suggest that many genes found in this region could be associated with AN. For instance, genetic variants associated with the serotonergic system (linked to the genes *HTR3A* and *HTR3B)* may be associated with the restrictive subtype of AN [49] and genetic variants of genes involved in the dopaminergic system (e.g., *DRD2*) might play a role in the susceptibility for AN in some populations [50, 51]. Interestingly the caudate is a brain structure that is strongly modulated by ascending dopaminergic projections [52] and implicated in reward processing [53, 54]. A number of PET studies indicate that aberrant striatal dopamine function may contribute to the behavioral phenotype in AN [55], although findings might be specific to the stage of recovery. For instance, the interaction between dopamine receptor and serotonin transporter binding was predictive of harm avoidance in recovered eating disorder patients [56, 57]. AN-related alterations in dopaminergic reward-related brain regions such as the caudate have also been shown using fMRI and monetary rewards, taste- or food-related stimuli [58]. However, the direction of change (increased or decreased activation) varied across studies [59–64].

Last, the genetic variant rs7945461—linked to *LRRC4C*, important in axonal and synaptic development [65, 66]—gained significance after conditioning on hippocampal volume. *LRRC4C* mRNA is abundant in hippocampal pyramidal neurons and dentate granule cells [67] and several studies have reported hippocampal volume reduction in acute AN patients relative to controls [7]. There has been no direct link so far between cognitive function and hippocampal alterations in AN [68], but there might be an indirect effect via estrogen-related hormone levels, which have been associated both with cognitive performance in AN [69] and with hippocampal volume regeneration upon weight restoration [14].

## Conclusion

In this comprehensive set of analyses, we found weak evidence for a relationship between common genetic variants implicated in AN and those associated with subcortical brain volumes at a high level (that is, common variant genetic architecture), but some suggestive evidence for effects of single genetic markers. Despite the sample sizes being the largest yet applied to these questions, more detailed multimodal brain- and genome-wide studies are needed to dissect the potential impact of genetic risk for AN on brain structure or function.

## Electronic Supplementary Material

ESM 1 (DOCX 773 kb)
