# Supplementary material for: Exploration of Shared Genetic Architecture Between Subcortical Brain Volumes and Anorexia Nervosa
Source: Mol Neurobiol. 2018 Dec 5;56(7):5146–56. doi: 10.1007/s12035-018-1439-4 (PMC6647452; doi:10.1007/s12035-018-1439-4)
Supplement: Supplementary file 1 — (DOCX 773 kb) [file 12035_2018_1439_MOESM1_ESM.docx]

Supplementary Material for:

Exploration of shared genetic architecture between subcortical brain volumes and Anorexia Nervosa

Walton E,^1,2^ Hibar D,^3,4^ Yilmaz Z,^5^ Jahanshad N,^3^ Cheung J,^3^ **Batury** VL,^2^ Seitz J,^6^ Bulik C,^5,7,8^, PGC-ED, ENIGMA Genetics Working Group, Thompson PM,^3^ Ehrlich S^2*^

^1^ MRC Integrative Epidemiology Unit, Population Health Sciences, Bristol Medical School, University of Bristol, Bristol, United Kingdom

^2^ Division of Psychological and Social Medicine and Developmental Neurosciences, Faculty of

Medicine, TU Dresden, Germany, Dresden, Germany

^3^ Imaging Genetics Center, Mark and Mary Stevens Neuroimaging & Informatics Institute, Keck

School of Medicine of the University of Southern California, Marina del Rey, CA, USA

^4^ Janssen Research & Development, San Diego, CA, USA

^5^ Department of Psychiatry, University of North Carolina at Chapel Hill, Chapel Hill, North Carolina

^6^ Department of Child and Adolescent Psychiatry, Psychotherapy and Psychosomatics, University Hospital RWTH University Aachen, Aachen, Germany

^7^ Department of Medical Epidemiology and Biostatistics, Karolinska Institutet, Stockholm, Sweden

^8^ Department of Nutrition, University of North Carolina at Chapel Hill, Chapel Hill, North Carolina

Corresponding author: Stefan Ehrlich, Division of Psychological and Social Medicine and Developmental Neurosciences, Faculty of Medicine, Technische Universität Dresden, Fetscherstr. 74, 01307 Dresden, Germany. E-mail: stefan.ehrlich@tu-dresden.de

SM 1 Methods

SM 1.1 ENIGMA

*Cohort description*

All participants in all cohorts in this study gave written informed consent and sites involved obtained approval from local research ethics committees or Institutional Review Boards. The ENIGMA consortium follows a rolling meta-analysis framework for incorporating sites into the analysis. The discovery sample comprises studies of European ancestry that contributed GWAS summary statistics for the purpose of this analysis on or before 1 October 2013. The deadline for discovery samples to upload their data was made before inspecting the data and was not influenced by the results of the analyses. The meta-analysis results from discovery cohorts were carried forward for secondary analyses and functional validation studies.

Supplementary Table S1: Demographic description of cohorts included in the ENIGMA analysis. Table adapted from Hibar et al. [1].

*Imaging measures*

The brain measures examined in this study were obtained from structural MRI data collected at participating sites around the world. Brain scans were processed and examined at each site locally, following a standardized protocol procedure to harmonize the analysis across sites. The standardized protocols for image analysis and quality assurance are openly available online (<http://enigma.ini.usc.edu/protocols/imagingprotocols/>). The subcortical brain measures (nucleus accumbens, amygdala, caudate, hippocampus, pallidum, putamen, and thalamus) were delineated in the brain using well-validated, freely available brain segmentation software packages: FIRST [2], part of the FMRIB Software Library (FSL), or FreeSurfer [3]. In addition to the subcortical structures of the brain, we examined the genetic effects of a measure of global head size, intracranial volume (ICV). After image processing, each image was inspected individually to identify poorly segmented structures. Each site contributed histograms of the distribution of volumes for the left and right hemisphere structures (and a measure of asymmetry) of each subcortical region used in the analysis. Scans marked as outliers (>3 standard deviations from the mean) based on the histogram plots were re-checked at each site to locate any errors. If a scan had an outlier for a given structure, but was segmented properly, it was retained in the analysis. Site-specific phenotype histograms, Manhattan plots, and QQ plots from each participating site are available on the ENIGMA website (<http://enigma.ini.usc.edu/publications/enigma-2/>).

*Genetic quality control (QC)*

Each study in the discovery sample was genotyped using commercially available platforms. Prior to imputation, genetic homogeneity was assessed in each sample using multi-dimensional scaling (MDS) analysis. Ancestry outliers were excluded through visual inspection of the first two components. Quality control filtering was applied to remove genotyped SNPs with low minor allele frequency (MAF < 0.01), poor genotype call rate ( < 95%), and deviations from Hardy-Weinberg Equilibrium (HWE p < 1*10-6) before imputation. Genomic data were imputed to a reference panel (1000 Genomes, v3 phase1) comprising only European samples and with monomorphic SNPs removed. The imputation protocols used MaCH [4] for haplotype phasing and minimac [5] for imputation and are freely available online (<http://enigma.ini.usc.edu/protocols/genetics-protocols/>). Only SNPs with an imputation score of RSQ > 0.5 and minor allele counts > 10 within each site were included. Tests of association were conducted separately for eight MRI volumetric phenotypes (amygdala, caudate, hippocampus, nucleus accumbens, pallidum, putamen, thalamus and intracranial volume (ICV)) with the following covariates in a multiple linear regression framework: age, age^2^, sex, four MDS components (to account for population structure), ICV (for analyses including subcortical brain phenotypes) and diagnosis (when applicable to case/control cohorts including patients with anxiety, Alzheimer’s disease, attention-deficit/hyperactivity disorder, bipolar disorder, epilepsy, major depressive disorder or schizophrenia).

*Statistical analysis*

Genome-wide association scans were conducted at each site for all eight traits of interest including ICV and bilateral volumes of the nucleus accumbens, amygdala, caudate, hippocampus, pallidum, putamen, and thalamus. For each SNP in the genome, the additive dosage value was regressed against the trait of interest separately using a multiple linear regression framework controlling for age, age^2^, sex, 4 MDS components, ICV (for non-ICV phenotypes), and diagnosis (when applicable). For studies with data collected from multiple centers or scanners, dummy-coded covariates were also included in the model. The resulting files were combined meta-analytically using a fixed-effect, inverse variance-weighted model as implemented in the software package METAL [6].

SM 1.2 Eating Disorder Working Group of the Psychiatric Genomics Consortium (PGC-ED)

*Cohort description*

PGC-ED is a collaboration representing researchers and clinicians from around the world, founded with the goal of identifying the genetic risk factors involved in the etiology of anorexia nervosa (AN). The Freeze 1 sample (2016) comprises 3,495 individuals with AN and 10,982 controls from 12 separate cohorts. Cases met DSM-IV criteria for either lifetime AN (restricting or binge-purge subtype) or lifetime eating disorders ‘not otherwise specified’ AN-subtype (i.e., exhibiting the core features of AN, also referred to as “atypical AN” ; ref [7]). Detailed information on recruitment and case ascertainment can be found elsewhere [8, 9]. Out of the 12 cohorts, the largest single contributor (1,031 cases and 3,627 controls post- QC) was the Children’s Hospital of Philadelphia/Price Foundation collection, and these samples were included in a previous AN GWAS publication [9]. The remaining 11 cohorts were wholly or in part from the Welcome Trust Case Control Consortium 3 (WTCCC3). The Welcome Trust Sanger Institute genotyped cases for all WTCCC3 AN samples as well as controls for two of the samples, and these cases and controls were included in a previous AN GWAS meta-analysis [8]. As the WTCCC3 did not fund genotyping of controls, ancestrally matched controls for 9 out of 11 WTCCC3 AN samples were sourced from multiple independent and overlapping research groups, consortia (including PGC), and funding bodies.

Supplementary Table S2: Descriptive information for 12 contributing PGC-ED studies. Case, control, and SNP numbers reflect final QC. Other than CHOP, all cases, plus cases and controls from Greece and Italy-South were from the Wellcome Trust Case Control Consortium 3 (WTCCC3). SNPs=single nucleotide polymorphisms, same=same chip as cases, CHOP=Children’s Hospital of Philadelphia, Czech Rep.=Czech Republic, Ill. Hum.660W=Illumina Human 660W-Quad, Ill.=Illumina. Table adapted from Duncan et al. [10].

*Genetic quality control (QC)*

Genotyping of cases and controls was performed using Illumina arrays (Illumina, Inc., San Diego, CA). QC was performed on each of the 12 individual datasets using the updated version of PLINK [11]. Exclusion criteria for SNP-level QC comprised: (1) missingness > 0.02; (2) minor allele frequency < 0.05; (3) differential missingness between cases and controls > 0.02; and (4) HWE cutoff of p < 1*10-6 for controls and p < 1*10-10 for cases. Exclusion criteria for sample-level QC comprised: (1) missingness > 0.02; (2) FHET > |0.2|; (3) failed sex check; and (4) π̂ > 0.2 for relatedness. Principal components analysis (PCA) was first performed within each dataset and then across all datasets using FastPCA [12], as implemented in the PGC pipeline [13].

*Statistical analysis/additional information*

Imputation to the 1000 Genomes phase 1 reference [14] was performed within the PGC pipeline using SHAPEIT [15] for phasing and IMPUTE2 [16] for imputation. Imputation was performed with a chunk size of 3Mb and using default parameters on the full set of 2,186 phased haplotypes (August 2012, 30,069,288 variants, release “v3.macGT1”). Analysis within datasets was performed using an additive model in PLINK, with the first ten principal components as covariates. Fixed-effects meta-analysis across the 12 datasets was accomplished using METAL, with inverse variance weighting. QC, imputation, and primary GWAS were performed following the Ricopili pipeline at the Broad Institute.

SM 2 Results

SM 2.1 Genetic risk score analysis

Supplementary Table S3: Detailed GRS results, carried out on subsets of four different thresholds (1*10^-4^, 1*10^-3^, 1*10^-2^, 5*10^-2^) in the discovery dataset (i.e. ENIGMA subcortical brain volume).

SM 2.2 Sign test

Supplementary Table S4: Detailed Sign test results, carried out on subsets of four different thresholds (1*10^-4^, 1*10^-3^, 1*10^-2^, 5*10^-2^) in the discovery dataset (i.e. ENIGMA subcortical brain volume **(grey) or AN (blue))**. ^a^ An estimate > 0.5 indicates a higher proportion of SNPs with a risk effect on AN (OR > 1) and on brain volume (BETA < 0)**. CI = confidence interval**

SM 2.3 Genomic location of rs3863294


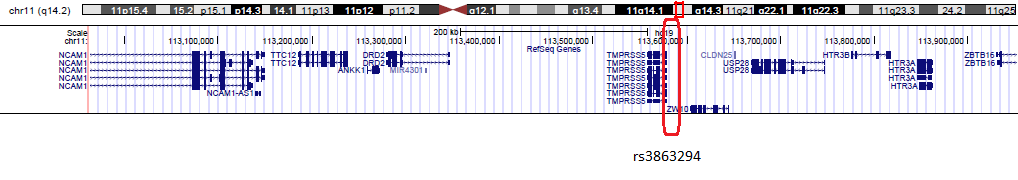


Supplementary Figure S1. Rs3863294, which was significantly associated with AN only after conditioning on its link to caudate volume, is found in the vicinity of neurotransmitter and immune system relevant genes such as *DRD2*, *HTR3B*, *HTR3A* and *NCAM.* Figure produced using the ucsc genome browser (GRCh38/hg38).

**SM 2.4 Functional effects of rs3863294 on DNA methylation and gene expression**

**We investigated rs3863294 SNP effects on methylation or (tissue-specific) gene expression to further investigate functional effects. For methylation-specific associations, we queried two publicly available online databases (mqtl.db.org based on blood samples and epigenetics.essex.ac.uk/mQTL based on fetal brain samples). We found that in blood rs3863294 appears to be an mQTL (in cis) for cg12522722, a methylation site associated with zw10 kinetochore protein (*ZW10*) involved in cell division (SM Figure S2). However, this association was not replicated in the fetal brain samples, and therefore, it remains unclear whether this SNP impacts DNA methylation in brain tissue.**

**
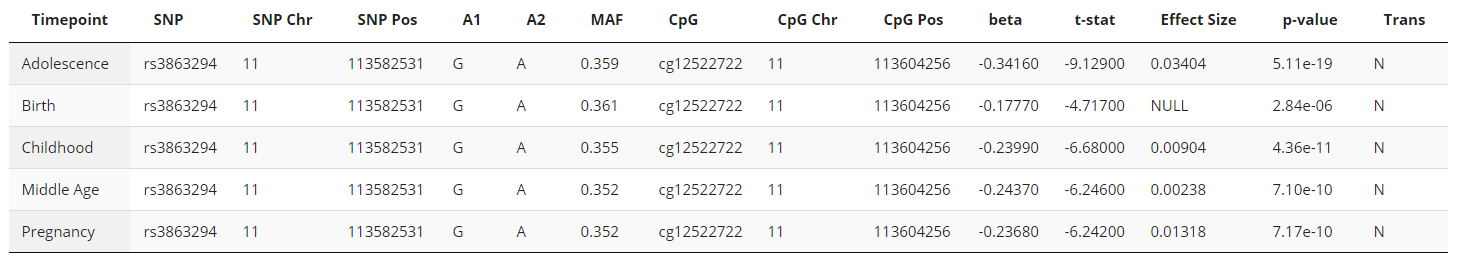
**

**SM Figure S2. mQTL query, investigating the effect of rs3863294 on DNA methylation in blood samples across the life course (mqtldb.org).**

**With respect to gene expression, we queried the GTEx resource (gtexportal.org) and found that rs3863294 does appear to impact *TMPRSS5* expression, predominantly in brain tissue. Strongest effects on expression were detected in subcortical brain structures, including the caudate (SM Figure S3). rs3863294 was also linked to *DRD2* expression, albeit most prominently so in skin tissue (SM Figure S4).**

**SM Figure S3. Effect of rs3863294 on *TMPRSS5* expression across tissues (based on GTEx data).**

**SM Figure S4. Effect of rs3863294 on *DRD2* expression across tissues (based on GTEx data).**

**SM 2.5 SNP Effect Concordance Analysis (SECA): Conditional analyses**


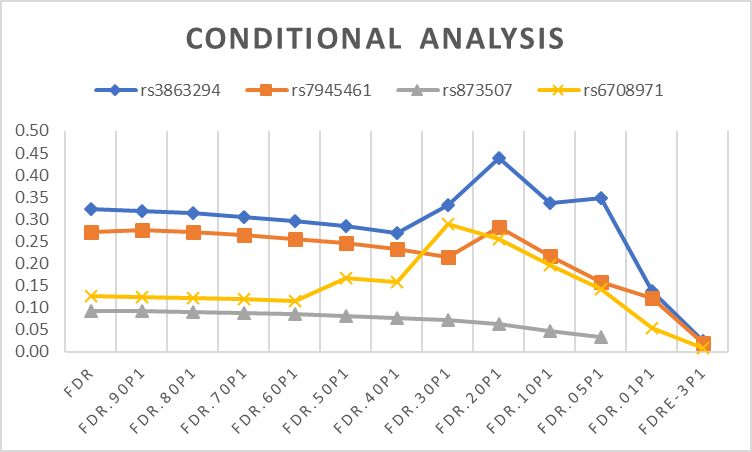


**Supplementary Figure S5. Conditional effects for four SNPs (differently colored lines), reported to gain in significance with AN (y-axis) after conditioning on brain volume (related to the caudate (blue), hippocampus (orange), pallidum (grey) and accumbens (yellow); x-axis). All effects were specific to more stringent FDR-corrected p-values (in the GWAS on brain volumes, x-axis) with a decrease in AN-significance at moderately stringent cutoffs (0.4 > p_FDR_ > 0.05). Only the SNP associated with effects conditional on pallidum volume (rs873507) showed a continuous gain in significance with AN, especially at p-value thresholds of 0.1 and 0.5 (note that analyses for the last two thresholds (p=0.01 and 0.003) did not converge).**

SM 2.6 Mendelian randomization analysis

| exposure | outcome | SNP | b | se | p |
| --- | --- | --- | --- | --- | --- |
| Caudate | AN | rs1318862 | -5.95E-04 | 1.11E-03 | 0.592 |
| Hippocampus | AN | rs61921502 | 2.16E-04 | 1.11E-03 | 0.846 |
| Hippocampus | AN | rs77956314 | -1.66E-04 | 9.86E-04 | 0.866 |
| Putamen | AN | rs6087771 | 1.80E-04 | 9.44E-04 | 0.849 |
| Putamen | AN | rs62097986 | 2.40E-04 | 9.78E-04 | 0.806 |
| Putamen | AN | rs683250 | -1.37E-03 | 1.09E-03 | 0.209 |
| ICV | AN | rs17689882 | -5.23E-07 | 2.66E-06 | 0.844 |

Supplementary Table S5. Mendelian randomization analysis to investigate a causal effect of brain volume on AN. Only brain volumes with genetic variants associated at a genome-wide level were investigated (caudate (1 SNP), hippocampus (2 SNPs), putamen (4 SNPs, of which 3 were available in the AN GWAS summary data), and ICV (1 SNP); as reported in Hibar et al. [1]).

References

1. Hibar DP, Stein JL, Renteria ME, et al (2015) Common genetic variants influence human subcortical brain structures. Nature 520:224–229 . doi: 10.1038/nature14101

2. Patenaude B, Smith SM, Kennedy DN, Jenkinson M (2011) A Bayesian model of shape and appearance for subcortical brain segmentation. NeuroImage 56:907–922 . doi: 10.1016/j.neuroimage.2011.02.046

3. Fischl B, Salat DH, Busa E, et al (2002) Whole Brain Segmentation: Automated Labeling of Neuroanatomical Structures in the Human Brain. Neuron 33:341–355 . doi: 10.1016/S0896-6273(02)00569-X

4. Li Y, Willer CJ, Ding J, et al (2010) MaCH: Using Sequence and Genotype Data to Estimate Haplotypes and Unobserved Genotypes. Genet Epidemiol 34:816–834 . doi: 10.1002/gepi.20533

5. Howie B, Fuchsberger C, Stephens M, et al (2012) Fast and accurate genotype imputation in genome-wide association studies through pre-phasing. Nat Genet 44:955–959 . doi: 10.1038/ng.2354

6. Willer CJ, Li Y, Abecasis GR (2010) METAL: fast and efficient meta-analysis of genomewide association scans. Bioinformatics 26:2190–2191 . doi: 10.1093/bioinformatics/btq340

7. American Psychiatric Association (1995) Diagnostic and statistical manual of mental disorders / DSM-IV, 4. ed., 4. pr. American Psychiatric Association

8. Boraska V, Franklin CS, Floyd J a. B, et al (2014) A genome-wide association study of anorexia nervosa. Mol Psychiatry 19:1085–1094 . doi: 10.1038/mp.2013.187

9. Wang K, Zhang H, Bloss CS, et al (2011) A genome-wide association study on common SNPs and rare CNVs in anorexia nervosa. Mol Psychiatry 16:949–959 . doi: 10.1038/mp.2010.107

10. Duncan L, Yilmaz Z, Walters R, et al (2016) Genome-Wide Association Study Reveals First Locus for Anorexia Nervosa and Metabolic Correlations. bioRxiv 088815 . doi: 10.1101/088815

11. Purcell S, Neale B, Todd-Brown K, et al (2007) PLINK: A Tool Set for Whole-Genome Association and Population-Based Linkage Analyses. Am J Hum Genet 81:559–575

12. Galinsky KJ, Bhatia G, Loh P-R, et al (2016) Fast Principal-Component Analysis Reveals Convergent Evolution of ADH1B in Europe and East Asia. Am J Hum Genet 98:456–472 . doi: 10.1016/j.ajhg.2015.12.022

13. Schizophrenia Working Group of the Psychiatric Genomics Consortium (2014) Biological insights from 108 schizophrenia-associated genetic loci. Nature 511:421–427 . doi: 10.1038/nature13595

14. 1000 Genomes Project Consortium, Abecasis GR, Altshuler D, et al (2010) A map of human genome variation from population-scale sequencing. Nature 467:1061–1073 . doi: 10.1038/nature09534

15. Delaneau O, Marchini J, Zagury J-F (2012) A linear complexity phasing method for thousands of genomes. Nat Methods 9:179–181 . doi: 10.1038/nmeth.1785

16. Howie BN, Donnelly P, Marchini J (2009) A flexible and accurate genotype imputation method for the next generation of genome-wide association studies. PLoS Genet 5:e1000529 . doi: 10.1371/journal.pgen.1000529
